# Uterine PEComa With Lymphangioleiomyomatosis (LAM)‐Like Features: A Case Report

**DOI:** 10.1155/crip/1620175

**Published:** 2026-02-25

**Authors:** Ramani Raman, Daisy Maharjan, Carina Dehner, Sheila Segura

**Affiliations:** ^1^ Department of Pathology and Laboratory Medicine, Indiana University School of Medicine, Indianapolis, Indiana, USA, indiana.edu; ^2^ Department of Pathology and Laboratory Medicine, Hospital of the University of Pennsylvania, Philadelphia, Pennsylvania, USA, pennmedicine.org

**Keywords:** lymphangioleiomyomatosis, PEComas, TSC1, uterus

## Abstract

Perivascular epithelioid cell tumors (PEComas) of the uterus are rare mesenchymal neoplasms characterized by myogenic and melanocytic differentiation. These tumors can mimic other uterine mesenchymal tumors in their clinical presentation and morphology. We report a case of a 58‐year‐old woman who presented with abnormal uterine bleeding. Initial imaging revealed an endometrial polyp, which was later diagnosed as a leiomyoma on excision biopsy. A subsequent hysterectomy with bilateral salpingectomy revealed a uterine neoplasm composed of spindled to epithelioid cells with low‐grade cytologic atypia, infiltrative edges, and extensive lymphovascular invasions, initially suggestive of low‐grade endometrial stromal sarcoma. However, immunohistochemical stains demonstrated tumor positivity for HMB45, desmin, and Cathepsin K, and genetic testing revealed a *TSC1* variant, leading to a definite diagnosis of uterine PEComa with lymphangioleiomyomatosis (LAM)‐like features. The patient′s postoperative course was uneventful, and follow‐up imaging showed no evidence of metastatic disease. This case highlights the importance of integrated morphological, immunohistochemical, and molecular assessment in differentiating uterine mesenchymal neoplasms to guide appropriate clinical management.

## 1. Introduction

Perivascular epithelioid cell tumors (PEComas) represent a rare subtype of mesenchymal neoplasms composed of perivascular epithelioid cells that exhibit both myogenic and melanocytic differentiation [1, 2]. The PEComa family encompasses a broad spectrum of tumors, including renal angiomyolipoma (AML), clear cell “sugar” tumor of the lung, primary extrapulmonary “sugar” tumor, lymphangioleiomyomatosis (LAM), clear cell myomelanocytic tumor, and primary cutaneous PEComa [3]. These tumors exhibit a strong female predominance, with a female‐to‐male ratio of approximately 4:1 [3]. Notably, around 25% of all PEComas arise within the female genital tract, most frequently involving the uterine corpus [2].

There is a well‐established association between PEComas and tuberous sclerosis complex (TSC), with inactivating mutations in *TSC1* or *TSC2* genes frequently identified in these tumors [4]. Additionally, a distinct molecular subtype of PEComa characterized by *TFE3* gene rearrangements has been described. These rearrangements are mutually exclusive with *TSC* mutations and show a separate pathogenic mechanism [5].

A particularly rare variant known as LAM‐like PEComa has been described [6]. These tumors closely resemble pulmonary LAM in morphology and are mostly found in the uterus, retroperitoneum, or pelvis, predominantly affecting women. Histologically, they feature bundles of spindle cells infiltrating around vessels, lymphatics, and smooth muscle layers, mimicking the architectural pattern seen in LAM. Accurate diagnosis of this uncommon subtype requires a high index of suspicion and correlation with immunohistochemistry and molecular findings [6].

## 2. Materials and Methods

We report a case of uterine PEComa with LAM‐like features. Histopathological, immunohistochemical, and molecular studies were performed and compared with findings reported in existing literature. A systematic literature review was conducted using databases (PubMed, Web of Science, and Google Scholar) with a combination of the following search terms: PEComas, uterine PEComas, and PEComas with LAM‐like features. Only articles in the English language were included.

### 2.1. Clinical Presentation

A 58‐year‐old woman presented with severe postmenopausal bleeding and anemia. Transabdominal and pelvic ultrasonography revealed multiple uterine fibroids and an endometrial polyp, which was excised and histologically identified as a leiomyoma. Despite initial intervention, she continued to experience persistent vaginal bleeding.

Subsequent transvaginal ultrasound demonstrated a heterogeneous endometrial thickening measuring 16 mm and a cervical mass up to 4.2 cm with internal vascularity. She underwent a laparoscopic hysterectomy with bilateral salpingectomy. Intraoperative findings included a distorted uterus and a large intracavitary mass protruding through the cervical os.

Initial histopathological examination of the uterine specimen suggested low‐grade endometrial stromal sarcoma (LG‐ESS). The specimen was referred to our institution for further evaluation.

### 2.2. Pathologic Findings

#### 2.2.1. Macroscopic Findings

Two specimens were submitted for pathologic evaluation.

Specimen 1: An intracavitary uterine mass, received as fragmented, irregular pink‐tan rubbery tissue measuring 6.0 × 4.0 × 1.5 cm in aggregates. Due to fragmentation, its site of origin could not be determined.

Specimen 2: A uterus with attached cervix and bilateral fallopian tube segments. The uterus weighed 132 g and measured 10.0 × 5.5 × 5.0 cm. The cervix and fallopian tubes were grossly unremarkable. A 3.9 cm polypoid lesion was present on the posterior endometrial wall. The myometrium measured up to 2.2 cm in thickness, with tan, glistening, friable tissue seen in dilated spaces in the posterior myometrial wall, suggesting possible vascular involvement.

#### 2.2.2. Microscopic Findings

Histopathological examination showed sheets of tumor cells with irregular and infiltrative borders extending into the surrounding myometrium (Figure 1a). Extensive lymphovascular invasion (LVI) with lobules of tumor inside vessels within the myometrium (Figure 1b) and foci of necrosis were noted. The tumor cells ranged from spindled to epithelioid in morphology, with scant cytoplasm, round to ovoid nuclei, and small nucleoli (Figure 1c). A prominent area with perivascular distribution of the spindled to ovoid tumor cells was observed, along with cleft‐like spaces and scattered thick‐walled vessels (Figure 1d). Mitotic activity was infrequent, with fewer than 1 mitotic figure per 50 high‐power fields (HPFs).

Figure 1(a) Tumor showing infiltrating edge into adjacent myometrium (hematoxylin and eosin, 200×). (b) Low‐power view showing lobules of tumor cells within lymphovascular spaces (hematoxylin and eosin, 40×). (c) Tumor cells exhibit spindled to epithelioid morphology with scant cytoplasm, round to ovoid nuclei, and small nucleoli (hematoxylin and eosin, 400×). (d) Lymphangioleiomyomatosis‐like pattern characterized by spindled to ovoid cells, cleft‐like spaces, and thick‐walled blood vessels (hematoxylin and eosin, 200×). (e) Desmin immunostaining shows strong and diffuse positivity in tumor cells (200×). (f) HMB45 shows strong focal staining in perivascular tumor cells (400×). (g) Cathepsin K reveals weak and focal staining in tumor cells near blood vessels (400×). (h) CD10 shows negative staining in tumor cells (400×).

**Figure:**
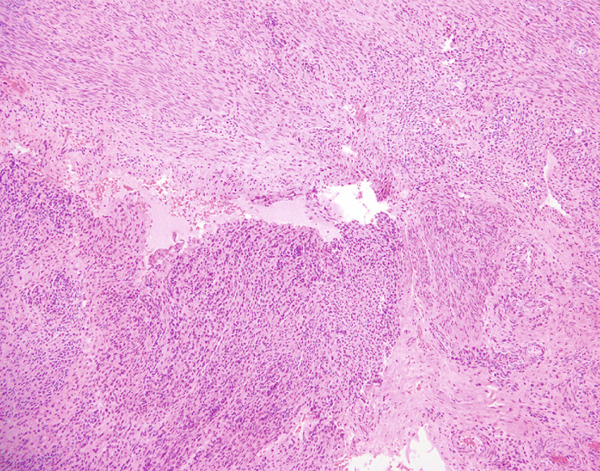
(a)

**Figure:**
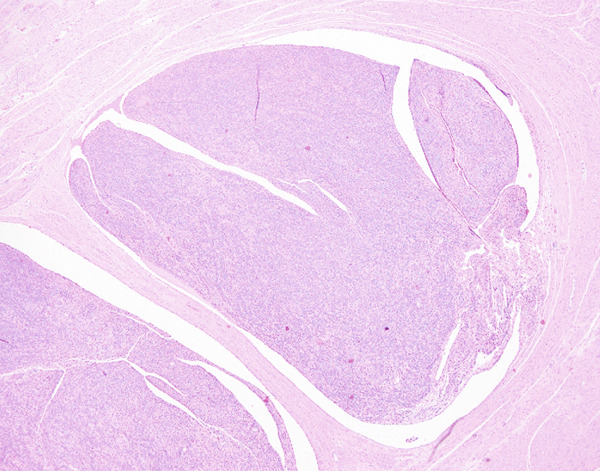
(b)

**Figure:**
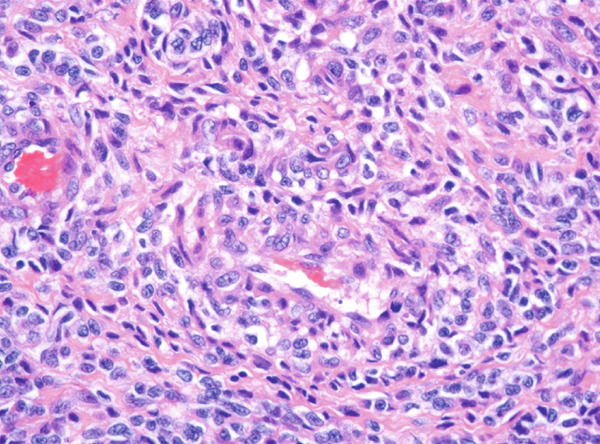
(c)

**Figure:**
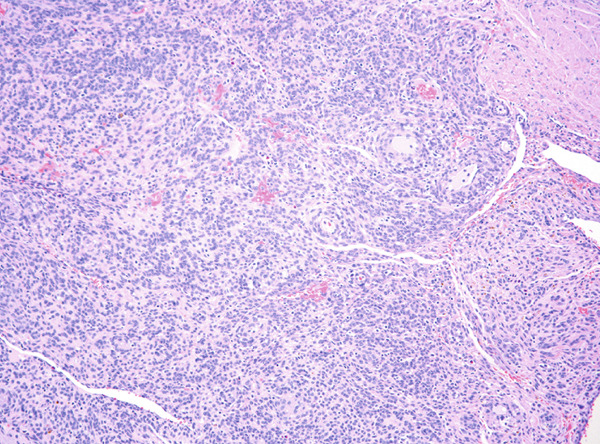
(d)

**Figure:**
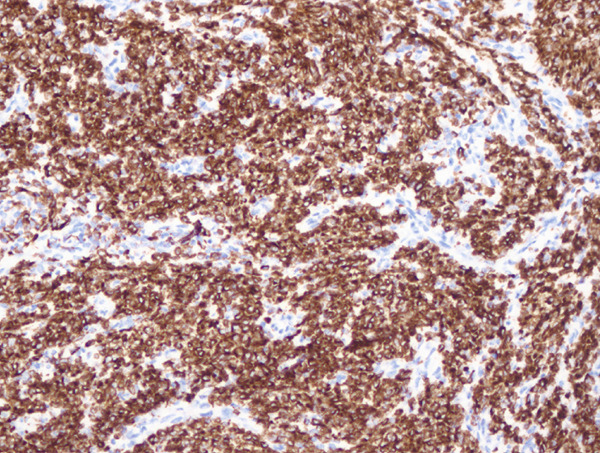
(e)

**Figure:**
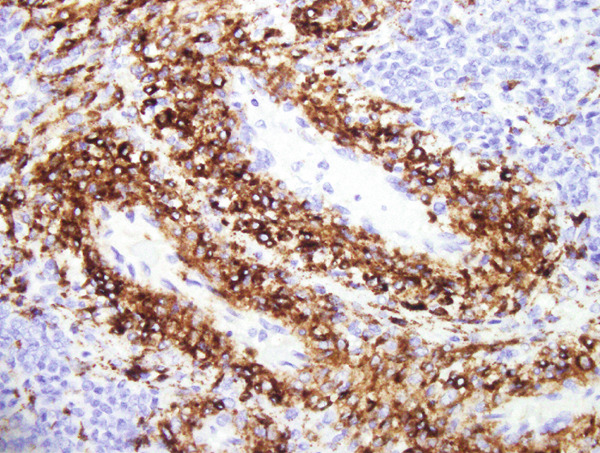
(f)

**Figure:**
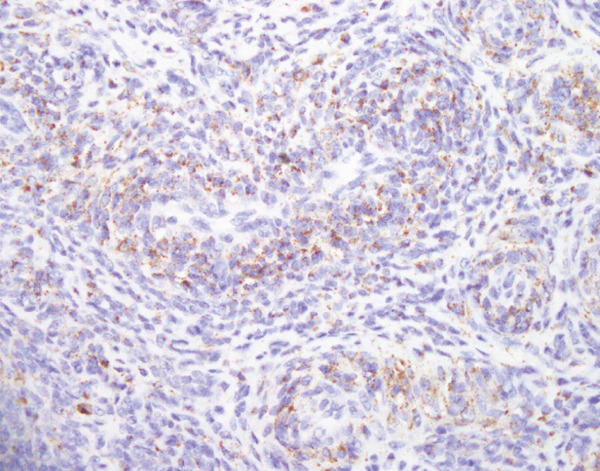
(g)

**Figure:**
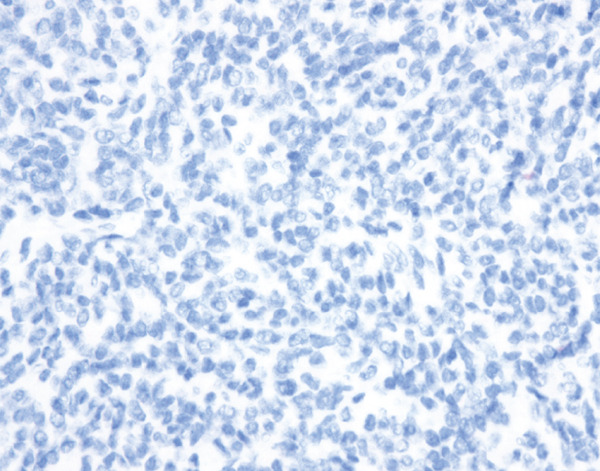
(h)

#### 2.2.3. Immunohistochemistry

Immunohistochemical staining showed strong and diffuse positivity for desmin (Figure 1e) and smooth muscle actin. The tumor also demonstrated focal positivity for HMB45 (Figure 1f) and Cathepsin K (Figure 1g) in the perivascular tumor cells. Estrogen receptor (ER) and progesterone receptor (PR) were expressed in approximately 70% and 30% of tumor cells, respectively. The tumor was negative for caldesmon, Melan‐A, MiTF, tyrosinase, CK AE1/3, CD10 (Figure 1h), CD34, and Cyclin D1.

#### 2.2.4. Molecular Findings

Formalin‐fixed, paraffin‐embedded tumor tissue was submitted to Caris Life Sciences for *MI Cancer Seek*, a next‐generation sequencing (NGS)–based single‐site assay. This platform evaluates single‐nucleotide variants (SNVs), insertions, and deletions across 228 cancer‐related genes, as well as microsatellite instability and tumor mutational burden [7].

A pathogenic frameshift mutation was detected in Exon 15 of the *TSC1* gene. Specifically, a DNA alteration c.1637_1657del17 was detected, resulting in a protein change p.D546fs. The variant allele frequency was 87%, indicating a high tumor cell fraction harboring this mutation.

The patient′s postoperative recovery was uncomplicated. Follow‐up PET‐CT imaging conducted at 2 and 6 months postoperatively revealed no evidence of metastasis.

## 3. Discussion

PEComas are rare mesenchymal neoplasms characterized by distinct histological and immunohistochemical features, including coexpression of melanocytic and smooth muscle markers. Approximately 25% of all PEComas arise within the female genital tract, with the uterine corpus being the most frequently affected site [2]. A review of 58 case reports of gynecological PEComa comprising 84 patients revealed a median age of 45 years (range 11–80 years) with 81% of cases involving the uterus [8]. A small number of cases involving the cervix, adnexa, vagina, broad ligament, and vulva have been reported [9].

Uterine PEComas typically present with nonspecific symptoms such as abnormal bleeding, pelvic pain, or palpable masses. They may mimic fibroids or polyps and are occasionally found incidentally during evaluation for adnexal masses or surgeries for presumed leiomyomas. Grossly, these tumors typically present as well‐demarcated intramural masses with solid, pink‐tan to white cut surfaces. A subset can show hemorrhage and necrosis, particularly in tumors with malignant potential [10].

Histologically, PEComas may exhibit well‐circumscribed or infiltrative borders, with sheets and nests of predominantly epithelioid cells and occasional spindle cells. The cytoplasm ranges from clear to granular eosinophilic, reflecting dual melanocytic and smooth muscle differentiation [3, 11]. The background vasculature typically consists of thin, delicate vessels, although thick‐walled vessels can be present at the periphery [10]. Cytologic atypia and mitotic activity vary and play a role in determining malignant potential. Other features may include prominent melanoma‐like nucleoli, intranuclear pseudoinclusions, multinucleated giant cells, Touton‐like giant cells, and rare melanin pigment [10].

Given their rarity and overlapping features with other neoplasms, PEComas pose a diagnostic challenge. Differential diagnoses include smooth muscle tumors, endometrial stromal sarcomas, melanoma (both primary and metastatic), and alveolar soft part sarcoma (ASPS) [11]. In our case, the infiltrative myometrial neoplasm composed of spindled to epithelioid cells with low‐grade atypia, perivascular distribution, and extensive LVI initially suggested LG‐ESS.

LG‐ESS can present as a polypoid mass or as soft, yellow, tan to white intramural nodules. It typically demonstrates myometrial invasion or LVI with jagged infiltrative borders, mild nuclear atypia, and a low mitotic index. Its morphology often resembles that of proliferative phase endometrium [12].

Distinguishing PEComas from smooth muscle tumors based solely on morphology is challenging. However, vascular and cytoplasmic features can aid differentiation. PEComas often display delicate, capillary‐like vasculature surrounding the tumor cells and nests, whereas smooth muscle tumors exhibit thick‐walled vessels, perinuclear vacuolization, and nongranular eosinophilic cytoplasm [6]. ASPS may share prominent perivascular architecture similar to PEComas but is distinguished by PAS‐positive intracytoplasmic rod‐shaped crystals.

On immunohistochemistry, PEComas typically express both melanocytic and myogenic markers. Among melanocytic markers, HMB45 is the most sensitive (92% positivity), followed by MiTF (83%) and Melan‐A (67%) [3]. Cathepsin K often shows strong, diffuse staining, while PNL2 displays variable cytoplasmic expression [3]. Common myogenic markers include desmin (variable ~80%), SMA (~88%), and caldesmon (variable ~76%) [10]. Hormone receptors are variably expressed, with ER reported to be positive in 53% and PR in 85% of cases [13].

PEComas typically lack expression of CD10, S100, and SOX10. In contrast, LG‐ESS often shows positivity for CD10, ER, PR, and androgen receptor, with desmin positivity in select cases and negative HMB45. Smooth muscle tumors are generally negative for melanocytic markers, although rare cases may express Cathepsin K [6]. ASPS is typically negative for HMB45 and Melan‐A and shows variable staining for desmin (see Table 1).

**Table 1:** Common immunohistochemical and molecular findings of PEComa and its differentials.

Immunohistochemistry	PEComa	Uterine smooth muscle tumors	Low‐grade endometrial stromal sarcoma (LG‐ESS)	Alveolar soft part sarcoma (ASPS)
Desmin	+	+	±	±
SMA	+	+	±	−
Caldesmon	+	+	±	−
HMB45	+	−	−	−
Melan‐A	+	−	−	−
MiTF	+	−	−	−
Cathepsin K	+	−	−	+
TFE3	+	−	−	+
ER	+	±	+	−
PR	+	±	+	−
CD10	−	−	+	−
S100	−	−	−	−
CKAE1/AE3	−	−	−	−
Molecular profiles	TSC1/TSC2 mutations; TFE3, RAD51B gene rearrangements	Variable	*JAZF1::SUZ12*, *JAZF1::PHF1* fusions	*ASPSCR1::TFE3* fusion

*Note:* +, positive expression; ±, variable or focal expression; −, negative expression.

Abbreviations: ER, estrogen receptor; FH, fumarate hydratase; PR, progesterone receptor.

Molecularly, PEComas frequently harbor mutations in the *TSC1* (10%) and *TSC2* (80%) genes [4]. *TFE3* and *RAD51B* gene rearrangements have also been identified [6]. TFE3‐fusion PEComas tend to occur in younger individuals and lack *TSC1*/*TSC2* mutations [5]. Histologically, they exhibit nested or alveolar architecture with epithelioid cells containing clear to eosinophilic cytoplasm and strong nuclear TFE3 immunoreactivity. These tumors tend to behave more aggressively than conventional PEComas, with higher risks of recurrence and metastasis [14]. LG‐ESS, ASPS, and uterine smooth muscle tumors also have distinct molecular alterations, which can help differentiate them from PEComas (see Table 1).

In our case, the tumor showed diffuse positivity for desmin and SMA, with focal expression of HMB45 and Cathepsin K. Molecular analysis identified a TSC1 mutation. Additionally, histology revealed areas resembling LAM, characterized by spindled to epithelioid cells arranged around thick‐walled blood vessels with cleft‐like spaces. LAM‐like PEComas are very rare, with two cases reported by Benett et al., one associated with pulmonary LAM and the other with tuberous sclerosis [6]. Notably, our patient had no history of pulmonary LAM or tuberous sclerosis. The diagnosis of uterine PEComa with LAM‐like features was established through integration of histopathology, immunohistochemistry, and molecular findings.

There are multiple classification systems to predict the disease behavior of uterine PEComas based on histopathological features. Schoolmeester system classifies PEComas as benign or of uncertain malignant potential when fewer than four high‐risk features are present. These features include tumor size ≥ 5 cm, high‐grade nuclear atypia, mitotic activity > 1 per 50 HPFs, necrosis, and LVI. Tumors exhibiting four or more of these features are considered malignant [11].

A modified system by Bennett et al., adopted in the 2020 WHO Classification of Female Genital Tumors, recommends lowering the threshold for malignancy to three or more high‐risk features. Tumors with fewer than three features are categorized as benign or of uncertain malignant potential, with the latter term preferred due to the possibility of recurrence [6, 10, 15].

Modified Folpe criteria define malignancy as necrosis plus two or more features: size ≥ 5 cm, infiltrative growth, mitotic rate > 1/50 HPFs, LVI, or marked atypia [13].

In our case, the tumor was fragmented, measuring 6 cm in aggregate (Specimen 1) and 3.5 cm (Specimen 2). For classification purposes, the tumor size was considered to exceed 5 cm. According to Schoolmeester criteria, the presence of three high‐risk features (tumor size > 5 cm, necrosis, and LVI) places the tumor in the category of uncertain malignant potential. However, based on the WHO‐adopted Bennett criteria and the modified Folpe criteria, the tumor meets the diagnostic threshold for malignancy.

Surgical resection remains the primary treatment for uterine PEComas, similar to LG‐ESS. However, adjuvant therapy differs. LG‐ESS typically requires hormonal therapy, and in advanced or recurrent cases, chemotherapy with tyrosine kinase inhibitors may be considered. LG‐ESS carries a high risk of late recurrence, necessitating long‐term follow‐up [12].

Due to the rarity of PEComas, standardized treatment guidelines are lacking [8]. However, tumors harboring *TSC1* or *TSC2* mutations may benefit from targeted therapy with mTOR inhibitors such as sirolimus, which has been associated with improved survival outcomes [5]. PEComas with malignant potential can behave aggressively, with high rates of local recurrence and metastasis, commonly to the lungs [8]. Mezzapesa et al. reported a case of uterine PEComa initially misdiagnosed as epithelioid leiomyosarcoma, later presenting with axial skeletal metastases. Given this diagnostic challenge, which can delay appropriate treatment, PEComa should be considered in the differential diagnosis of uterine mesenchymal tumors with central pathology review recommended in suspected cases [16].

Follow‐up recommendation for malignant PEComas aligns with protocols for other uterine sarcomas, involving surveillance every 3–6 months during the first 5 years. Follow‐up PET imaging in our patient was done at 2 and 6 months and showed no evidence of metastatic disease.

## 4. Conclusions

The case highlights a rare morphological variant of uterine PEComa with LAM‐like features, presenting a diagnostic challenge due to its resemblance to LG‐ESS. It underscores the importance of integrating histopathology, immunohistochemistry, and molecular analysis for accurate diagnosis. Early identification and tailored therapy are essential for optimizing outcomes in patients with rare uterine mesenchymal tumors.

## Author Contributions

Concept, design, and coordination: S.S. and C.D.; compilation and analysis of clinicopathologic and clinical data: D.M. and R.R.; article draft, table, and figures: D.M., R.R., and S.S.; cases and/or intellectual contributions (including article editing): all authors.

## Funding

No funding was received for this manuscript.

## Consent

No written consent has been obtained from the patient as there is no patient‐identifiable data included in this case report.

## Conflicts of Interest

The authors declare no conflicts of interest.

## Data Availability

The data generated in this study are available from the corresponding author upon reasonable request.
